# Inhibition of Yes-Associated Protein by Verteporfin Ameliorates Unilateral Ureteral Obstruction-Induced Renal Tubulointerstitial Inflammation and Fibrosis

**DOI:** 10.3390/ijms21218184

**Published:** 2020-10-31

**Authors:** Jixiu Jin, Tian Wang, Woong Park, Wenjia Li, Won Kim, Sung Kwang Park, Kyung Pyo Kang

**Affiliations:** 1 Department of Internal Medicine, Research Institute of Clinical Medicine, Jeonbuk National University Medical School, Jeonju 54907, Korea; gilsoo1215@gmail.com (J.J.); tianw0000@outlook.com (T.W.); mnipw@hanmail.net (W.P.); liwenjia1214@gmail.com (W.L.); kwon@jbnu.ac.kr (W.K.); 2 Biomedical Research Institute, Jeonbuk National University Hospital, Jeonju 54907, Korea

**Keywords:** kidney fibrosis, inflammation, myofibroblast activation, extracellular matrix, Hippo pathway, verteporfin

## Abstract

Yes-associated protein (YAP) activation after acute ischemic kidney injury might be related to interstitial fibrosis and impaired renal tubular regeneration. Verteporfin (VP) is a photosensitizer used in photodynamic therapy to treat age-related macular degeneration. In cancer cells, VP inhibits TEA domain family member (TEAD)-YAP interactions without light stimulation. The protective role of VP in unilateral ureteral obstruction (UUO)-induced renal fibrosis and related mechanisms remains unclear. In this study, we investigate the protective effects of VP on UUO-induced renal tubulointerstitial inflammation and fibrosis and its regulation of the transforming growth factor-β1 (TGF-β1)/Smad signaling pathway. We find that VP decreased the UUO-induced increase in tubular injury, inflammation, and extracellular matrix deposition in mice. VP also decreased myofibroblast activation and proliferation in UUO kidneys and NRK-49F cells by modulating Smad2 and Smad3 phosphorylation. Therefore, YAP inhibition might have beneficial effects on UUO-induced tubulointerstitial inflammation and fibrosis by regulating the TGF-β1/Smad signaling pathway.

## 1. Introduction

Chronic kidney disease (CKD) is a heterogeneous condition characterized by reduced glomerular filtration rate, glomerular sclerosis, tubular atrophy, and interstitial fibrosis with inflammatory cell infiltration [1]. As human life expectancy has increased, CKD has become one of the most common non-communicable diseases [2,3]. End-stage renal disease that requires renal replacement therapy such as dialysis or kidney transplantation is associated with a decrease in residual life expectancy compared with healthy individuals [3]. Despite plenty of health resources in developed countries, CKD’s global burden is steadily increasing, and CKD-related mortality is also increasing [4]. A nationwide cohort study in Korea found a higher mortality rate in patients with CKD than in healthy controls or even in patients with diabetes or hypertension but without CKD [5]. Therefore, strategies to enable the early recognition and prevention of CKD are needed to decrease the global health burden.

Organ fibrosis is characterized by a dynamic process of non-resolving inflammatory reactions [6] that cause the deterioration of organ function. After an ischemic or toxic injury to the kidneys causes damage to glomerular, vascular, and tubular cells, an inflammatory reaction can increase the deposition of the extracellular matrix (ECM) in the renal parenchyma [7]. Transforming growth factor-β1 (TGF-β1) is a multifunctional cytokine related to cell growth, differentiation, apoptosis, and wound repair [8]. Histologically, CKD is characterized by excessive ECM production from activated myofibroblasts, which causes renal tubulointerstitial fibrosis and organ dysfunction. TGF-β1 is a potent mediator in this fibrotic process [8,9]. Activated TGF-β1 binds to type I and type II cell surface receptors and phosphorylates the Smad2 and Smad3 proteins to activate the canonical (Smad-dependent) TGF-β signaling pathway [8,10]. TGF-β also activates other, non-canonical (Smad-independent) pathways, such as mitogen-activated protein kinase, phosphatidylinositol-3-kinase, and Rho-like GTPase [11]. Therefore, modulation of the TGF-β1 signaling pathway is an important therapeutic target for preventing progressive CKD. 

The Hippo pathway is an evolutionarily conserved signaling pathway that regulates cell growth and fate decisions, organ size control, and regeneration [12]. The Hippo pathway consists of a kinase cascade (mammalian Ste20-like kinase 1/2, large tumor suppressor 1/2, and downstream effectors and transcriptional coactivators), the Yes-associated protein (YAP), and a transcriptional coactivator with a PDZ-binding motif (TAZ) [13]. Once the Hippo pathway has been activated, it limits tissue growth and cell proliferation through the phosphorylation and degradation of YAP/TAZ. In contrast, when the Hippo pathway is turned off, YAP/TAZ is dephosphorylated and translocated into the nucleus to induce target gene transcription [12,13]. During kidney development, *Yap* and *Taz* activation produce different phenotypes. YAP is related to nephron morphogenesis, and TAZ inactivation causes polycystic kidney disease [14]. YAP/TAZ function as physical sensors of cell structure, shape, and polarity, and YAP/TAZ activation is related to mechanical signaling in cells involved in tissue architecture and the surrounding ECM [14]. Therefore, modulating YAP/TAZ might reveal novel therapeutic targets for preventing renal fibrosis.

Verteporfin (VP) is a photosensitizer already used in photodynamic therapy to treat age-related macular degeneration [15]. Recently, Liu-Chittenden et al. showed that VP inhibits TEA domain family member (TEAD)–YAP interactions in cancer cells without light stimulation [16]. Notably, connective tissue growth factor (CTGF) and TGF-β, which regulate the extent of remodeling in the tissue architecture, are among the YAP transcription targets [17]. Recently, YAP and CTGF were found to be closely involved in determining blood vessel integrity and stability in the retina [18]. The constant activation of YAP after acute ischemic kidney injury might be related to interstitial fibrosis and impaired renal tubular regeneration [19]. Therefore, using VP to inhibit YAP might be a novel treatment strategy for renal tubulointerstitial inflammation and fibrosis. However, the protective role of VP in unilateral ureteral obstruction (UUO)-induced renal fibrosis and the related mechanisms remain unclear. Therefore, in this study, we investigate the protective effects of VP on UUO-induced renal tubulointerstitial inflammation and fibrosis and its regulatory role in the TGF-β signaling pathway.

## 2. Results

### 2.1. Verteporfin Decreases UUO-Induced Renal Tubular Injury and Fibrosis

**Figure 1:**
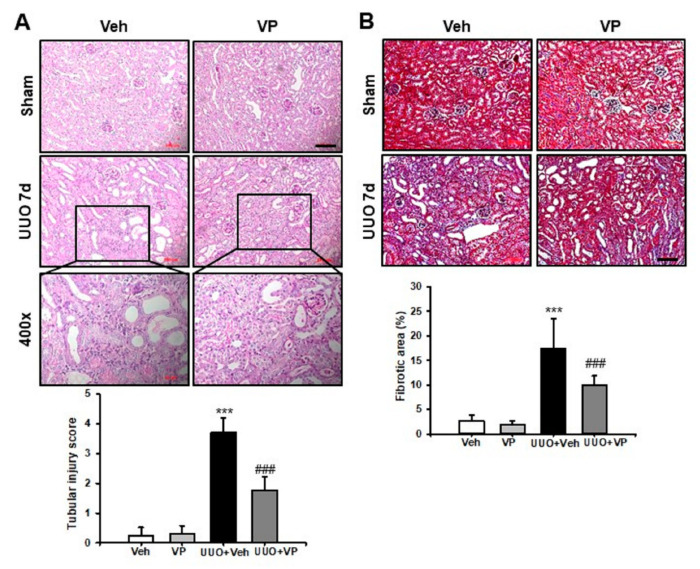
Effect of verteporfin on UUO-induced renal tubular injury and fibrosis. Representative PAS and MTC stains from the kidneys of sham- and UUO-operated mice treated with vehicle (Veh) or verteporfin (VP). Scale bar = 100 µm. Inlet shows a higher magnification view (400×) of UUO 7 d with Veh or VP treatment. The bar graphs show the semi-quantitative scoring of (**A**) tubular injury stained by PAS and (**B**) the area fraction (%) of tubulointerstitial fibrosis stained by MTC from ten randomly chosen, non-overlapping fields (*n* = 10) at a magnification of 200× (*n* = 15/group). Data are expressed as the mean ± SD. *** *p* < 0.001 versus Veh or VP; ^###^ *p* < 0.001 versus UUO. Veh, vehicle; VP, verteporfin; Sham, sham-operated mice; UUO, unilateral ureteral obstruction operated mice.

To investigate the effect of VP on UUO-induced renal tubular injury and fibrosis, we examined kidney sections after periodic acid–Schiff (PAS) and Masson’s trichrome staining. Histologically, Veh-treated UUO kidneys showed tubular dilation, epithelial desquamation, loss of brush border, inflammatory cell infiltration, and tubulointerstitial fibrosis. VP treatment decreased the UUO-induced increase in tubular dilation, inflammatory cell infiltration, and tubulointerstitial fibrosis compared with Veh-treated UUO kidneys (Figure 1A). Masson’s trichrome staining revealed an increase in the fibrotic areas of the Veh-treated UUO kidneys compared with sham-operated kidneys treated with either Veh or VP. VP treatment, on the other hand, significantly decreased the UUO-induced increase in fibrotic areas (Figure 1B). Those data suggest that VP treatment has a protective effect against UUO-induced tubular injury and fibrosis.

### 2.2. Verteporfin Decreases UUO-Induced Renal Fibroblast Activation and Excessive Extracellular Matrix Accumulation

**Figure 2:**
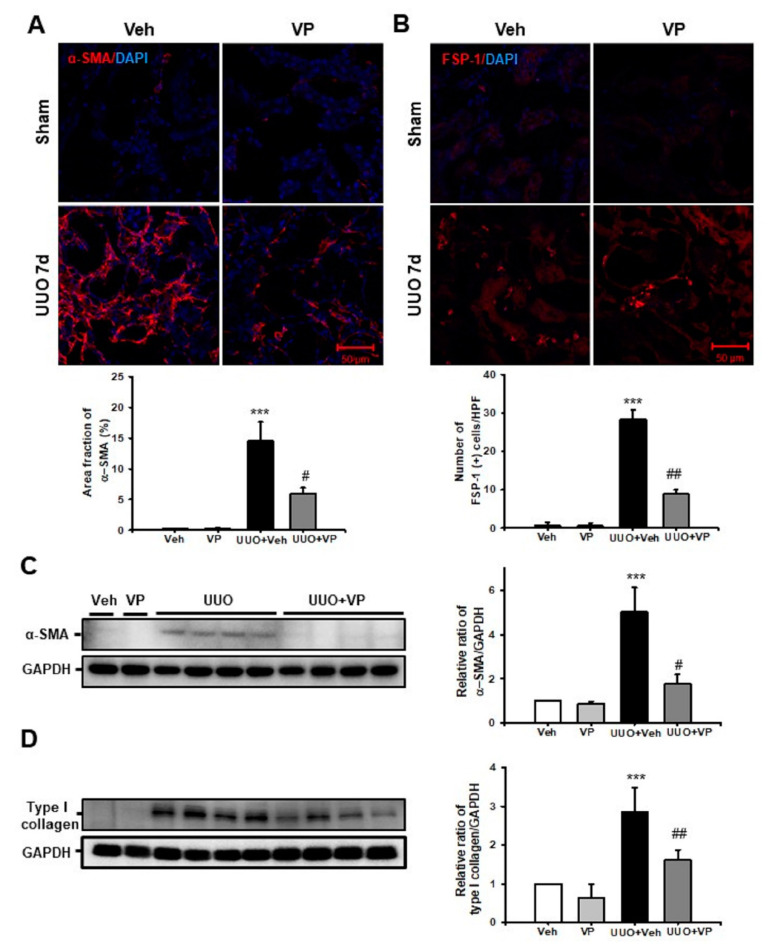
Effect of verteporfin on UUO-induced renal fibroblast activation. Representative immunofluorescence staining of α-SMA (**A**) and FPS-1 (**B**) from the kidneys of sham- and UUO-operated mice treated with vehicle (Veh) or verteporfin (VP). The nuclei were stained with DAPI. The bar graph shows the number of α-SMA and FPS-1 positive cells from ten randomly chosen, non-overlapping fields (*n* = 10) at a magnification of x400 (*n* = 15/group). Scale bar = 50 µm. Data are expressed as the mean ± SD. *** *p* < 0.001 versus Veh or VP; ^#^ *p* < 0.05, ^##^ *p* < 0.01 versus UUO. α-SMA (**C**) and Type I collagen (**D**) expression in kidney tissue from sham and UUO-operated mice treated with Veh or VP was evaluated by Western blotting. Data from the densitometric analysis are presented as the relative ratio of each protein to GAPDH. The relative ratio measured in the kidneys from sham-operated mice treated with Veh is arbitrarily presented as 1. Data are expressed as the mean ± SD. ^#^ *p* < 0.05 versus UUO. Veh, vehicle; VP, verteporfin; UUO, unilateral ureteral obstruction operated mice; α-SMA, α-smooth muscle actin; FSP-1; fibroblast-specific protein-1; DAPI, 4’,6-diamidino-2-phenylindole; GAPDH, glyceraldehyde 3-phosphate dehydrogenase.

Renal fibrosis is characterized by renal fibroblast activation and excessive ECM accumulation that leads to tissue destruction, scarring, and kidney failure [6,20,21]. Therefore, we investigated renal fibroblast activation after UUO surgery by examining α-Smooth muscle actin (α-SMA) and fibroblast specific protein-1 (FSP-1) expression. Veh-treated UUO kidneys showed an increase in the α-SMA-positive area compared with sham-operated kidneys that received Veh or VP treatment, and VP treatment significantly attenuated that increase in UUO kidneys (Figure 2A). We also evaluated α-SMA expression in a Western blot analysis of UUO kidneys with or without VP treatment. Veh-treated UUO kidneys had significantly increased α-SMA expression compared with sham-operated kidneys treated with Veh or VP, and VP treatment significantly decreased the UUO-induced increase in α-SMA expression (Figure 2C). After UUO surgery, the number of FSP-1 (+) fibroblasts increased significantly compared with sham-operated kidneys treated with either Veh or VP, and VP treatment significantly decreased the number of FSP-1 (+) fibroblasts in UUO kidneys (Figure 2B). We also evaluated type I collagen expression using Western blotting of the UUO kidneys. Type I collagen expression was increased in Veh-treated UUO kidneys compared with sham-operated kidneys, and VP treatment significantly decreased the UUO-induced increase in type I collagen expression (Figure 2D). These data suggest that VP treatment reduces UUO-induced renal fibroblast activation and EMC accumulation in the kidney.

### 2.3. Verteporfin Decreases UUO-Induced Renal Inflammation

**Figure 3:**
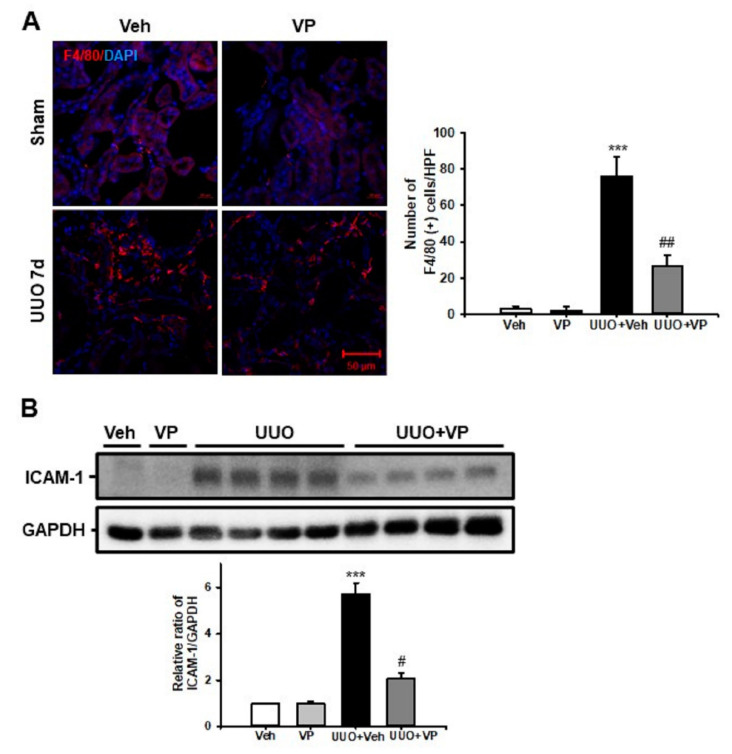
Effect of verteporfin on UUO-induced renal inflammation. (**A**) Representative immunofluorescence staining of F4/80 from the kidneys of sham- and UUO-operated mice treated with vehicle (Veh) or verteporfin (VP). The nuclei were stained with DAPI. The bar graph shows the number of F4/80 positive cells from ten randomly chosen, non-overlapping fields (*n* = 10) at a magnification of ×400 (*n* = 15/group). Scale bar = 50 µm. Data are expressed as the mean ± SD. *** *p* < 0.001 versus Veh or VP; ^##^ *p* < 0.01 versus UUO. (**B**) ICAM-1 expression in kidney tissue from sham- and UUO-operated mice treated with Veh or VP was evaluated by Western blotting. Data from the densitometric analysis are presented as the relative ratio of each protein to GAPDH. The relative ratio measured in the kidneys of sham-operated mice treated with Veh is arbitrarily presented as 1. Data are expressed as the mean ± SD. *** *p* < 0.001 versus Veh or VP; ^#^ *p* < 0.05 versus UUO; Veh, vehicle; VP, verteporfin; Sham, sham-operated mice; UUO, unilateral ureteral obstruction operated mice; ICAM-1, intercellular adhesion molecule-1; DAPI, 4’,6-diamidino-2-phenylindole; GAPDH, glyceraldehyde 3-phosphate dehydrogenase.

Renal inflammation, such as inflammatory cell infiltration and the increased expression of cell adhesion molecules, is an essential pathologic mechanism of UUO-induced renal tubulointerstitial fibrosis [21]. Therefore, we evaluated F4/80 (+) macrophage infiltration after UUO surgery and treatment with Veh or VP. Veh-treated UUO kidneys showed an increasing number of F4/80 (+) macrophages in the tubulointerstitial areas, and VP treatment significantly decreased the UUO-induced increase in F4/80 (+) macrophage infiltration (Figure 3A). We also used Western blot analysis to evaluate the expression of intercellular adhesion molecules (ICAM)-1 in UUO kidneys treated with Veh or VP. After UUO surgery, ICAM-1 expression increased, compared with sham-operated kidneys, and VP treatment significantly decreased the UUO-induced increase in ICAM-1 expression (Figure 3B). These data show that VP treatment reduces UUO-induced tubulointerstitial inflammation by regulating inflammatory cell infiltration and cell adhesion molecule expression. 

### 2.4. Verteporfin Decreases the UUO-Induced Increase in Connective Tissue Growth Factors by Regulating the Tgf-Β1/Smad Signaling Pathway

**Figure 4:**
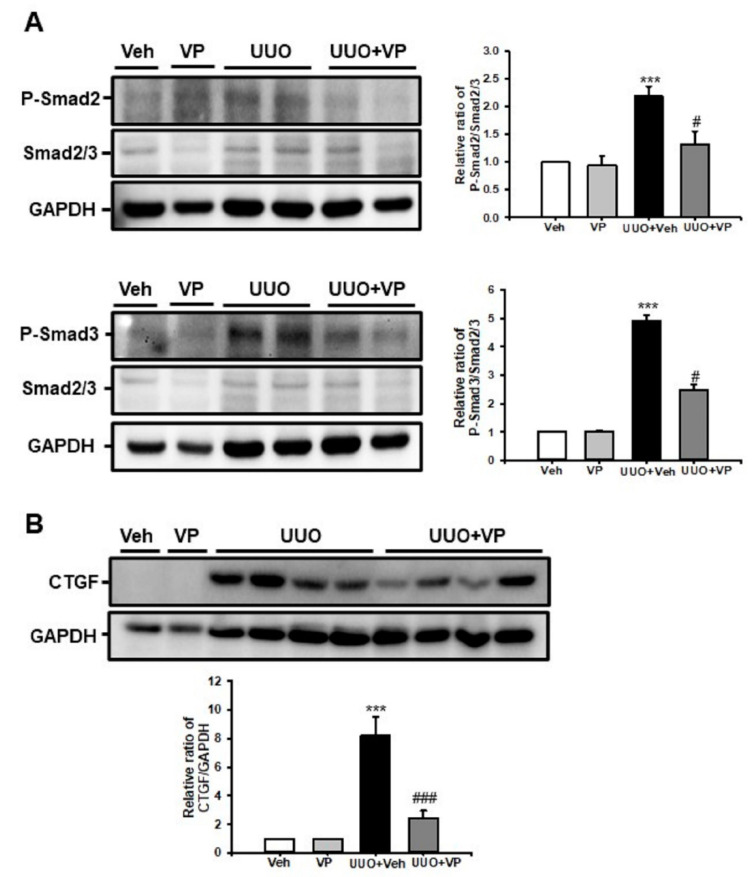
Effect of verteporfin on UUO-induced connective tissue growth factor expression through the regulation of Smad2 and Smad3 phosphorylation. (**A**) Phospho-Smad2 and phospho-Smad3 expression in kidney tissue from sham- and UUO-operated mice treated with vehicle (Veh) or verteporfin (VP) was evaluated by Western blotting. Data from the densitometric analysis of phospho-Smad2 and phospho-Smad3 are presented as the relative ratio of each protein to Smad2, Smad3, and GAPDH. The relative ratios measured in the kidneys of sham-operated mice treated with Veh are arbitrarily presented as 1. Data are expressed as the mean ± SD. *** *p* < 0.001 versus Veh or VP; ^#^ *p* < 0.05 versus UUO. (**B**) CTGF expression in kidney tissue from sham- and UUO-operated mice treated with Veh or VP was evaluated by Western blotting. Data from the densitometric analysis are presented as the relative ratio of each protein to GAPDH. The relative ratios measured in the kidneys of sham-operated mice treated with Veh are arbitrarily presented as 1. Data are expressed as the mean ± SD. *** *p* < 0.001 versus Veh or VP; ^###^ *p* < 0.001 versus UUO; P-Smad, phospho-Smad; CTGF, connective tissue growth factor; Veh, vehicle; VP, verteporfin; UUO, unilateral ureteral obstruction operated mice, GAPDH, glyceraldehyde 3-phosphate dehydrogenase.

To address the protective mechanism of VP in UUO-induced renal fibrosis, we evaluated the TGF-β1/Smad signaling pathway. UUO kidneys showed an increase in Smad2 and Smad3 phosphorylation compared with sham-operated kidneys treated with Veh or VP, and VP treatment significantly decreased the UUO-induced increase in Smad2 and Smad3 phosphorylation (Figure 4A). CTGF is a matricellular protein that has been associated with wound healing and organ fibrosis [22]. CTGF is known to be a downstream mediator of the profibrotic TGF-β1 signaling pathway [23]. Therefore, we evaluated CTGF expression by Western blot analysis. After UUO surgery, CTGF expression increased compared with sham-operated kidneys treated with Veh or VP, and VP treatment significantly decreased the UUO-induced increase in CTGF expression (Figure 4B). These data suggest that VP modulates the UUO-induced activation of the TGF-β1/Smad signaling pathway and the expression of CTGF in UUO kidneys. 

### 2.5. Verteporfin Decreases TGF-β1-Induced Renal Fibroblast Proliferation And Migration in NRK-49F Cells

**Figure 5:**
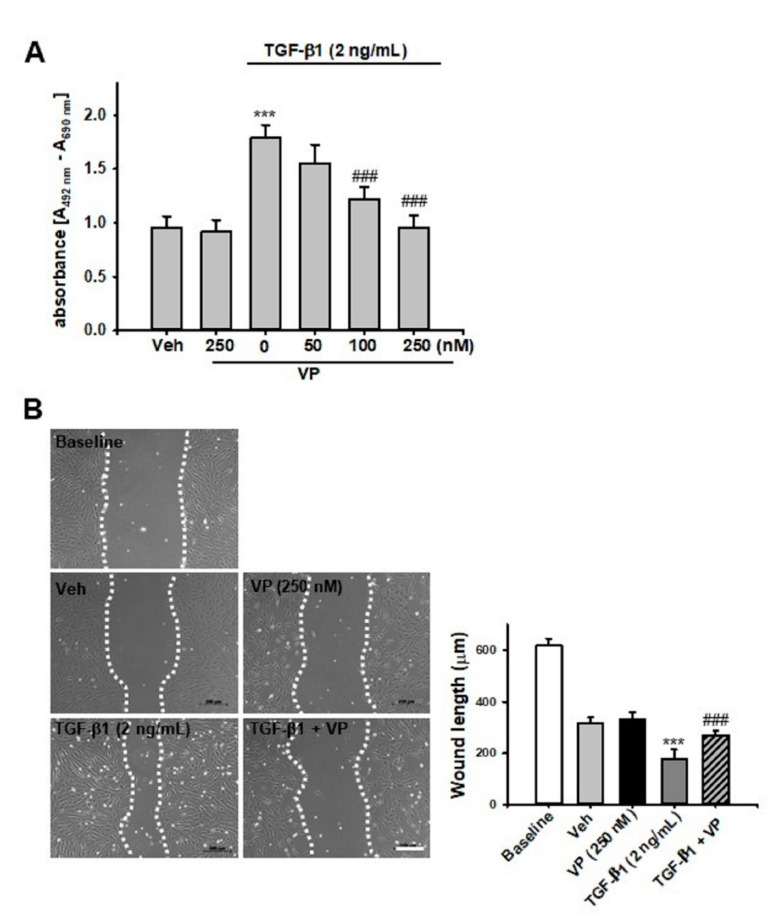
Effect of verteporfin on TGF-ß1-induced fibroblast proliferation and cell migration in NRK-49F cells. (**A**) NRK-49F cells were treated with vehicle (Veh) or TGF-β1 (2 ng/mL) with or without verteporfin (VP) at the indicated doses (50, 100, and 250 nM). After 24 h of treatment, cell proliferation was measured using the XTT assay. Data are expressed as the mean ± SD of three independent experiments performed in triplicate. *** *p* < 0.001 versus Veh or VP; ^###^ *p* < 0.001 versus TGF-β1 (2 ng/mL). (**B**) Representative phase-contrast images of NRK-49F cells after the wound healing assay. The phase-contrast images of the migration of NRK-49F cells into the scratch area were obtained after treatment with either Veh or TGF-β1 (2 ng/mL) with or without VP (250 nM) at 0 h and 24 h after wounding. The bar graph shows the average length by which the gap between the NRK-49F cells closed at 0 h or 24 h after treatment with Veh, TGF-β1, and/or VP. *** *p* < 0.001 versus Veh or VP; ^###^ *p* < 0.001 versus TGF-β1. Scale bar = 200 μm. Veh, vehicle; VP, verteporfin; TGF-β1, transforming growth factor-β1.

We evaluated the effect of VP on TGF-β1-induced renal fibroblast proliferation and migration in normal rat fibroblasts (NRK-49F cells). Treatment with TGF-β1 increased renal fibroblast proliferation about 1.8-fold compared with Veh-treated NRK-49F cells. VP treatment significantly and dose-dependently decreased the TGF-β1-induced increase in renal fibroblast proliferation (Figure 5A). We also evaluated cell migration using a wound-healing assay. After TGF-β1 treatment, wound length was significantly decreased compared with baseline and Veh-treated NRK-49F cells. VP treatment decreased the TGF-β1-induced increase in NRK-49F cell migration (Figure 5B). These data suggest that VP treatment regulates TGF-β1-induced renal fibroblast proliferation and migration in NRK-49F cells. 

### 2.6. Verteporfin Decreases TGF-β1-Induced Renal Fibroblast Activation by Regulating the TGF-β1/Smad Signaling Pathway in NRK-49F Cells

**Figure 6:**
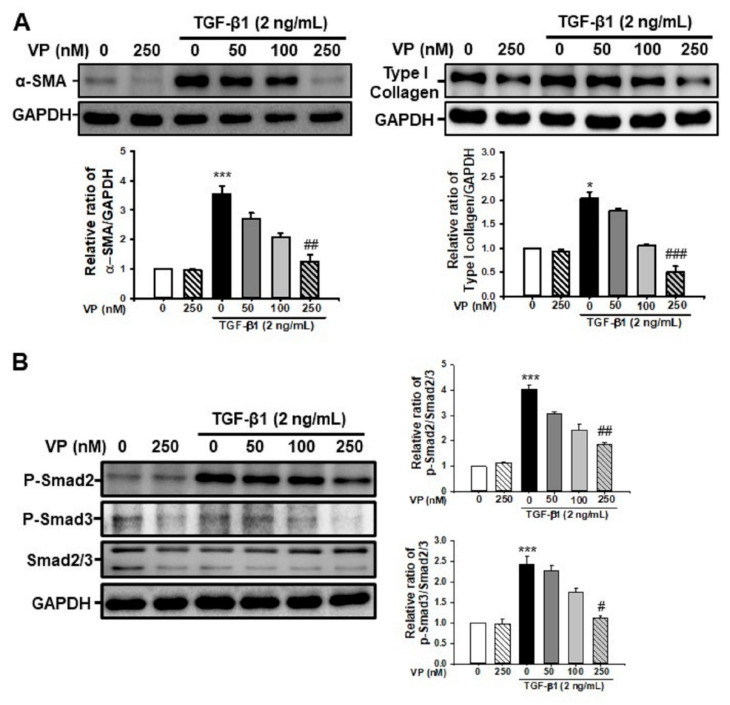
Effect of verteporfin on TGF-ß1-induced α-SMA and type I collagen through the regulation of the TGF-β1/Smad signaling pathway in NRK-49F cells. (**A**) Representative Western blot for α-SMA and type I collagen from NRK-49F cells treated with vehicle (Veh) or TGF-β1 (2 ng/mL) with or without verteporfin (VP) at the indicated doses (50, 100 and 250 nM). Treatment with TGF-β1 (2 ng/mL) for 24 h increased the expression of fibrotic markers. The expression of α-SMA and type I collagen decreased dose-dependently after VP treatment. The bar graph shows the densitometric quantification as the relative ratio of each protein to GAPDH. Data are presented as the mean ± SD. (**B**) Representative Western blot for phospho-Smad2 and phospho-Smad3 expression from NRK-49F cells treated with vehicle (Veh) or TGF-β1 (2 ng/mL), with or without VP at the indicated doses (50, 100, and 250 nM). TGF-β1 (2 ng/mL) treatment for 30 min increased the expression of phospho-Smad2 and phospho-Smad3, and VP treatment dose-dependently decreased that increase. The densitometric measurement of phospho-Smad2 and phospho-Smad3 protein expression is presented as the Smad2/3 protein expression ratio. The data are represented as the mean ± SD. *** *p* < 0.001 versus Veh or VP; ^#^ *p* < 0.05, ^##^ *p* < 0.01 versus TGF-β1 treatment. Veh, vehicle; VP, verteporfin; TGF-β1, transforming growth factor-β1; α-SMA, α-smooth muscle actin; GAPDH, glyceraldehyde 3-phosphate dehydrogenase.

We evaluated whether VP could modulate renal myofibroblast activation and matrix protein production in NFK49F cells. After 24 h of stimulation, TGF-β1 (2 ng/mL) significantly increased α-SMA and type I collagen expression in NRK-49F cells, and VP treatment significantly and dose-dependently decreased the TGF-β1-induced increase in α-SMA and type I collagen expression (Figure 6A). We further evaluated the effect of VP on the TGF-β1/Smad signaling pathway in NRK-49F cells. After 30 min of stimulation, TGF-β1 (2 ng/mL) increased Smad2 and Smad3 phosphorylation in NRK-49F cells, and VP treatment significantly and dose-dependently decreased the TGF-β1-induced increase in Smad2 and Smad3phosphorylation (Figure 6B). These data suggest that VP modulates TGF-β1-induced renal fibroblast activation through the TGF-β1/Smad signaling pathway.

## 3. Discussion

Renal fibrosis is a common pathophysiologic endpoint of advanced CKD. The loss of the glomerular and peritubular capillary architecture, the proliferation of tubular cells and interstitial fibroblasts, the increases in proinflammatory cytokines and chemokines, the infiltration of inflammatory cells, and the diffuse accumulation of ECM are common histologic features in renal fibrosis [24]. Previously, we found that inhibiting the TGF/Smad signaling pathway by modulating estrogen receptor α and activating mitochondrial Sirt3 ameliorated UUO-induced renal inflammation and fibrosis [9,21]. In this study, we evaluated the protective effect of VP on UUO-induced renal fibrosis. Our results indicate that VP treatment decreases UUO-induced renal tubular injury, ECM deposition, and inflammatory processes. VP also inhibits TGF-β1-induced renal fibroblast activation. These results show that VP has a protective effect against kidney fibrosis by regulating the TGF-β1/Smad signaling pathway. 

The excessive accumulation of ECM protein drives progressive organ fibrosis, which increases tissue stiffness and activates the mechanosensitive Hippo pathway effector YAP [25]. Active YAP upregulates ECM deposition and activates a positive-feedback loop that results in fibroblast activation [26]. Therefore, reducing ECM accumulation by modulating YAP activity is a critical target for preventing organ fibrosis and failure. In this study, we used a UUO model to induce renal fibrosis, which produced an increase in the number of FSP-1 (+) fibroblasts and α-SMA (+) myofibroblasts. VP treatment decreased the UUO-induced increase in fibroblast activation. VP treatment also decreased the UUO-induced increase in type I collagen and fibronectin expression. Therefore, using VP treatment to inhibit the Hippo pathway effector YAP might decrease UUO-induced ECM accumulation.

To initiate the wound healing process, the recruitment of inflammatory cells is a fundamental process [27]. The infiltration of polymorphonuclear neutrophils and monocytes is characteristic of the initial inflammatory response to injury [28,29]. An injured organ’s parenchymal and endothelial cells and infiltrated inflammatory cells release many cytokines and chemokines that amplify the inflammatory response through complex interactions among the altered mesenchymal cells [27]. Dysregulation of the inflammatory and wound-healing processes leads to the formation of tissue fibrosis. In this study, VP treatment decreased the UUO-induced increase in F4/80 (+) macrophage infiltration and cell adhesion molecule expression. These data suggest that inhibiting the Hippo pathway might decrease the UUO-induced renal inflammatory response.

The TGF-β1/Smad signaling pathway is a crucial player in renal fibrosis [9,20]. The downstream target genes of the TGF-β1/Smad signaling pathway mediate myofibroblast activation and ECM deposition in injured tissues. Our in vitro data using NFR49F cells show that inhibiting the Hippo pathway decreases TGF-β1-induced renal fibroblast proliferation and migration by regulating Smad2 and Smad3 phosphorylation. Furthermore, VP treatment reduces TGF-β1-induced ECM production in NRK-49F cells. The action of TGF-β1 and YAP activity might by closely related at multiple levels of the fibrotic process, such as tissue stiffness by ECM accumulation, cell adhesion, and cell morphology [30]. In human lung fibrosis, increased TGF-β1 and aberrantly activated TAZ/YAP can contribute to fibroblast activation and survival and enhance the production of profibrogenic factors such as CTGF [30]. In diabetic nephropathy, EGF receptor-dependent upregulation of YAP increases the levels of downstream profibrotic factors such as CTGF and amphiregulin [31]. TGF-β1-dependent TAZ activation promotes maladaptive epithelial repair through Smad3-dependent CTGF up-regulation [32]. Xu et al. reported that constant YAP increases and activation are involved in regeneration and fibrogenesis after acute ischemic kidney injury [19]. In our in vivo experiment, the inhibition of YAP by VP decreased the UUO-induced increase in CTGF expression by regulating Smad2 and Smad3 phosphorylation. 

The Hippo pathway plays an essential role in kidney and urinary tract development [33,34], cystic kidney disease [35,36], podocyte integrity [37], diabetic nephropathy [31], renal cell carcinoma [38], and tubulointerstitial fibrosis [39]. In the Hippo pathway, both YAP and TAZ can serve as central transcriptional coactivators after nuclear translocation. They associate with transcription factors such as the Runt-related transcription factor and TEAD to modulate transcription [40,41,42,43]. Recent research data have linked YAP and TAZ with fibrogenesis as critical regulators of fibroblast mechanoactivation and fibrogenic function [26]. Renal fibrosis that arises from numerous insults progresses to CKD, which is characterized by the deposition of ECM. The stiff ECM enhances TGF-β1-induced profibrotic Smad signaling in a process mediated by YAP and TAZ [44]. In addition to ECM deposition, YAP has an essential role in glomerular integrity. Podocyte-specific *Yap* deletion results in proteinuria between 5 and 6 weeks of age and leads to focal segmental glomerular sclerosis at 12 weeks [37]. Therefore, the modulation of the Hippo pathway might be a novel therapeutic target for kidney fibrosis.

In conclusion, YAP inhibition by means of VP treatment decreases UUO-induced renal fibroblast activation, inflammation, ECM deposition, and tubulointerstitial fibrosis by regulating the TGF-β1/Smad signaling pathway.

## 4. Materials and Methods

### 4.1. Animal Experiment

The animal experiment protocol was reviewed and approved by the Institutional Animal Care and Use Committee of Jeonbuk National University (CBNU 2018-040, 29 May 2018, Jeonju, Korea). Male C57BL/6 mice (7–8 weeks old; weight 20–25 g) were purchased from Orient Bio, Inc. (Seoul, Korea), maintained in a room with controlled temperature (23 ± 1 °C), humidity, and lighting (12-h light/12-h dark cycle), and given free access to food and water. For the experiment, we divided the mice into four groups: sham and UUO with Veh treatment, and sham and UUO with VP treatment (*n* = 15/group). VP (Sigma-Aldrich; Merck KGaA, Darmstadt, Germany) was dissolved in dimethyl sulfoxide (DMSO, 0.05% *v*/*v*), and DMSO (0.05% *v*/*v*) was used as the vehicle. VP (100 mg/kg) was administered by daily intraperitoneal injection for 3 d before UUO surgery and continued for 7 d after surgery. Renal fibrosis was induced using a UUO operation described previously [21]. In brief, mice were anesthetized by intraperitoneal injection of ketamine (100 mg/kg, Huons, Seoul, Korea) and xylazine (10 mg/kg, Bayer Korea, Seoul, Korea) and placed on a temperature-controlled operating table to maintain body temperature at 37 °C. Through a midline incision in the abdomen, the right proximal ureter was exposed and ligated at two separated points using 3-0 black silk. We performed the sham operation using the same method without the ligation of the ureter. Seven days after the UUO operation, the obstructed kidney was harvested, prepared for histological examination, and stored at −80 °C for the Western blot analysis.

### 4.2. Renal Histologic Examination

Each kidney was fixed in 4% paraformaldehyde and embedded in paraffin. The block was cut into 5 μm sections and stained with PAS stain and Masson’s trichrome. For immunofluorescence staining, freshly frozen renal tissues were fixed with 4% paraformaldehyde, permeabilized in 1% Triton X-100, and then incubated with a blocking buffer. The tissue samples were incubated with anti-α-smooth muscle actin (α-SMA; A2547 mouse; 1:1000; Sigma-Aldrich Merck KGaA, Darmstadt, Germany), anti-fibroblast specific protein (FSP)-1 (ab27957; 1:100; Abcam, Cambridge, UK) and F4/80 (14-4801-82; 1:200 eBioscience, San Diego, CA, USA) and then exposed to Cy3-labeled secondary antibody (Chemicon, Temecula, CA). Nuclear staining was performed using 300 nM 4’, 6-diamidino-2-phenylindole solution for 3 min (DAPI, Molecular Probes; Thermo Fisher Scientific, Inc.). For the morphometric analysis, two observers who were unaware of the origins of the samples used a Zeiss Z1 microscope or Zeiss LSM 510 confocal laser scanning microscope (Carl Zeiss, Göttingen, Germany) to evaluate all slides. Tubular injury was scored into six levels based on the percentage of tubular dilation, epithelial desquamation, and loss of brush border in 10 randomly chosen, non-overlapping fields at a magnification of 200× under a light microscope: 0, none; 0.5, <10%; 1, 10–25%; 2, 25–50%; 3, 50–75%; and 4, >75%. The fibrotic area was also measured in 10 randomly chosen, non-overlapping fields at a magnification of 200×. The area fraction of α-SMA was measured at a magnification of 400×. The numbers of FSP-1 positive fibroblasts and F4/80 positive macrophages were counted at a magnification of 400×. All images were analyzed using ImageJ software (http://rsb.info.nih.gov/ij).

### 4.3. Western Blotting

The Western blot analysis was performed as described previously [20]. Kidney tissue and cell lysates were separated by 10% SDS-PAGE. After electrophoresis, the samples were transferred to PVDF membranes (BIO-RAD, Hercules, CA, USA) and blocked with 5% skim milk (BIO-RAD, Hercules, CA, USA). Then we probed the blots with primary antibodies to α-SMA (A2547 mouse; 1:1000; Sigma-Aldrich Merck KGaA, Darmstadt, Germany), type I collagen (1310-01; goat; 1:1000; Southern Biotech, Birmingham, AL, USA), ICAM-1 (sc-1511; goat; 1:1000, Santa Cruz Biotechnology, Mississauga, CA, USA), CTGF (sc-365970; mouse; 1:1000; Santa Cruz Biotechnology, Inc., Dallas, TX, USA), phospho-Smad2 (3101; rabbit; 1:1000; Cell Signaling Technology Inc., Danvers, MA, USA), phospho-Smad3 (9520; rabbit; 1:1000; Cell Signaling Technology Inc.), Smad2/3 (07-408; rabbit; 1:1000; EMD Millipore, Billerica, MA, USA), and glyceraldehyde 3-phosphate dehydrogenase (GAPDH; AP0063; rabbit; 1:2000; Bioworld Technology, Inc., Danvers, MA, USA), which was used as an internal control. All signals were analyzed by a densitometric scanner (ImageQuant LAS 4000 Mini, GE Healthcare Life Sciences, Piscataway Township, NJ, USA).

### 4.4. Cell Culture Experiments

In vitro experiments were performed using a rat renal fibroblast cell line (NRK-49F, American Type Culture Collection, Manassas, VA). We cultured NRK-49F cells in Dulbecco’s modified Eagle’s medium with 4 mM L-glutamine adjusted to contain 1.5 g/L of sodium bicarbonate and 4.5 g/L of glucose supplemented with 5% (vol/vol) heat-inactivated fetal bovine serum and antibiotics (100 U/mL penicillin G and 100 μg/mL streptomycin) at 37 °C with 5% CO_2_ in 95% air. To investigate the effect of VP on myofibroblast activation, ECM expression, and the activation of the TGF-β1/Smad signaling pathway, we incubated sub-confluent NRK-49F cells with VP (50, 100, and 250 nM) for 30 min and then stimulated them with TGF-β1 (2 ng/mL, Sigma Chemical Co.) for the indicated periods.

### 4.5. Cell proliferation Assay

After 24 h of treatment with VP (50, 100, and 250 nM) and TGF-β1 (2 ng/mL), the proliferation of NRK-49F cells was determined by a colorimetric assay (Cell Proliferation Kit II, Roche Diagnostics, Mannheim, Germany) according to the manufacturer’s protocol. All experimental values were determined from triplicate wells.

### 4.6. Wound Healing Assay

Sub-confluent NRK-49F cells were cultured in 6-well dishes. Before treatment with VP and TGF-β1, we scratched the 6-well dishes using a sterile 200-μL pipette tip, causing three separate wounds. The cells were incubated with VP (250 nM) for 30 min and then stimulated with TGF-β1 (2 ng/mL) for 24 h. Wound lengths were measured using ImageJ. The wound length at 0 h after scratching was used as the control.

### 4.7. Statistical Analysis

The data are expressed as the mean ± standard deviation (SD). To confirm whether the dataset was normally distributed, we used the Shapiro-Wilk test. For normally distributed data, one-way analysis of variance (ANOVA) was used to evaluate differences within groups, followed by an individual comparison between groups with the Tukey post hoc test. For non-parametric data, the Kruskal-Wallis one-way ANOVA on ranks was used, followed by all multiple pairwise comparisons with the Dunn’s method. *p* < 0.05 was considered statistically significant.

## Abbreviations

α-SMA	α-smooth muscle actin
CKD	chronic kidney disease
CTGF	connective tissue growth factor
DAPI	4’,6-diamidino-2-phenylindole
ECM,	extracellular matrix
FSP-1	fibroblast-specific protein-1
GAPDH	glyceraldehyde 3-phosphate dehydrogenase
ICAM-1	intercellular adhesion molecule-1
MTC	Masson’s trichrome.
NRK	normal rat kidney
PAS	periodic acid–Schiff
Sham	sham-operated mice
TAZ	transcriptional coactivator with a PDZ-binding motif
TEAD	TEA domain family member
TGF-β1	transforming growth factor-β1
UUO	unilateral ureteral obstruction
YAP	Yes-associated protein
Veh	Vehicle
VP	Verteporfin
